# Progressive dyspnea and diffuse ground‐glass opacities after treatment for lymphoma with rituximab‐containing chemotherapy: A case report

**DOI:** 10.1111/1759-7714.13473

**Published:** 2020-05-06

**Authors:** Yuxin Sun, Chi Shao, Kai Xu, Ji Li, Ying Zhang, Peng Liu, Hui Huang, Ruie Feng

**Affiliations:** ^1^ Department of Pulmonary and Critical Care Medicine Peking Union Medical College Hospital, Chinese Academy of Medical Sciences & Peking Union Medical College Beijing China; ^2^ Radiological Department Peking Union Medical College Hospital, Chinese Academy of Medical Sciences & Peking Union Medical College Beijing China; ^3^ Pathological Department Peking Union Medical College Hospital, Chinese Academy of Medical Sciences & Peking Union Medical College Beijing China; ^4^ International Medical Service Department Peking Union Medical College Hospital, Chinese Academy of Medical Sciences & Peking Union Medical College Beijing China; ^5^ Medical Research Center, Central Laboratory Peking Union Medical College Hospital, Chinese Academy of Medical Sciences & Peking Union Medical College Beijing China

**Keywords:** Drug associated lung disease, ground‐glass opacities, lymphoma, rituximab

## Abstract

A 49‐year‐old man presented to our outpatient clinic complaining of nonproductive cough and exertional dyspnea for two months. He had been diagnosed with large B cell non‐Hodgkin's lymphoma seven months previously, and the tumor had almost disappeared after four cycles of rituximab‐containing chemotherapy. He then developed a severe dry cough, progressive dyspnea and hypoxia two weeks after the fifth cycle. Bilateral diffuse ground‐glass opacities were visible on chest X‐ray. Although the patient's symptoms were ameliorated temporarily after two weeks of methylprednisolone administration and multiple antibiotics, exertional dyspnea had progressed slowly starting one month after discontinuation of the corticosteroid. A repeat chest computed tomography (CT) scan showed diffuse ground‐glass opacities, bronchoalveolar lavage fluid tests for pathogens were negative and the pathological manifestation of the transbronchial lung biopsy showed nonspecific interstitial pneumonia. Rituximab‐induced interstitial lung disease was diagnosed after multidisciplinary discussion. Prednisone was again prescribed and his symptoms and the pulmonary opacities gradually disappeared. Although various pulmonary infections are the most common respiratory complications in patients with non‐Hodgkin's lymphoma undergoing rituximab‐containing chemotherapy, noninfectious diffuse lung disease, eg, drug‐associated interstitial lung disease might be considered as a differential diagnosis of patients treated with rituximab, especially if a patient is nearing the time of administration of a fourth cycle of rituximab.

## Introduction

Various pulmonary infections are the most common respiratory complications for patients with non‐Hodgkin's lymphoma undergoing rituximab‐containing chemotherapy. However, noninfectious diffuse lung disease might also be life‐threatening. Here, we report the case of a patient with large B cell non‐Hodgkin's lymphoma who developed progressive dyspnea and diffuse ground‐glass opacities (GGOs) two weeks after a fifth cycle of rituximab‐containing chemotherapy.

## Case report

**Figure 1:**
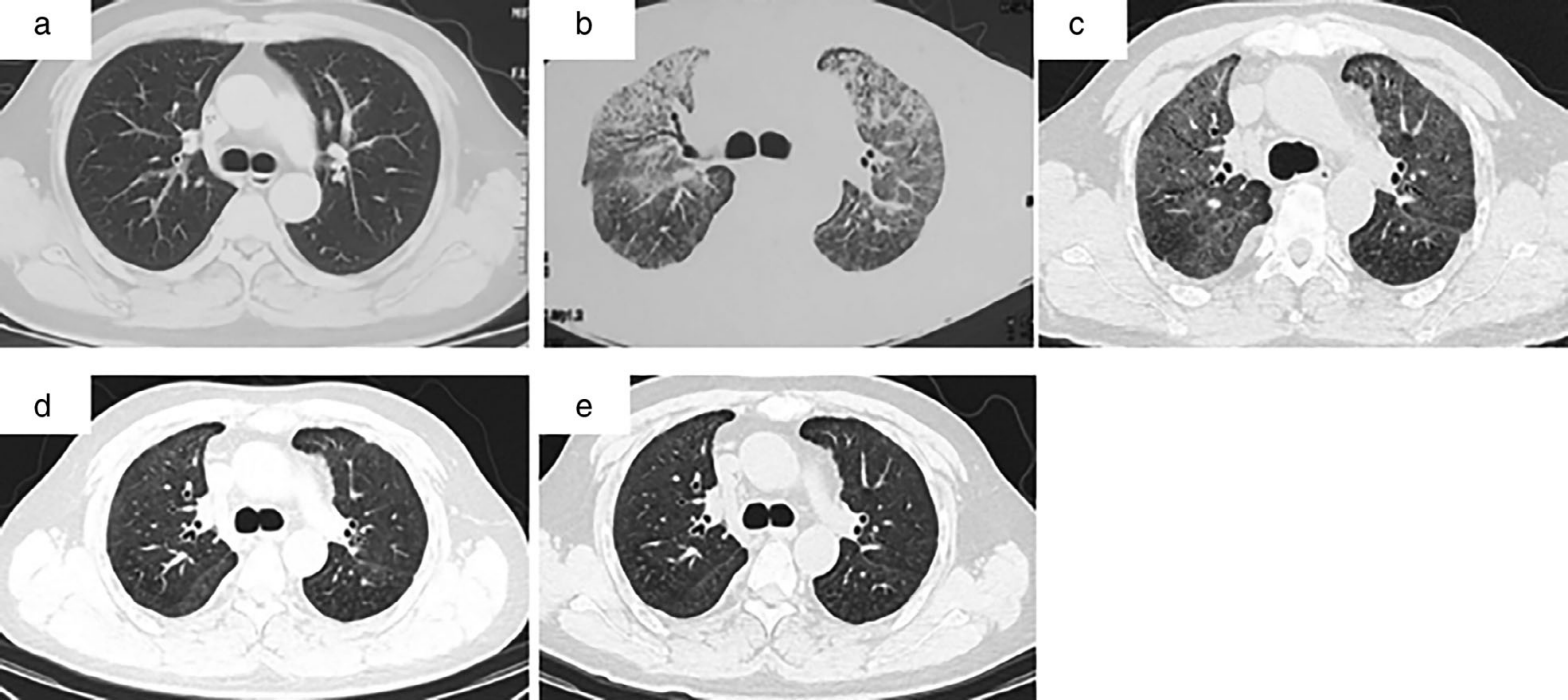
Series chest computed tomography (CT) imaging during the treatment. (**a**) There was no significant abnormal opacities visible on chest CT in February 2018. (**b**) The repeat high‐resolution CT (HRCT) scan of his chest in June 2018 showed diffuse bilateral GGOs without obvious lymphadenopathy. (**c**–**e**) The pulmonary GGOs gradually disappeared on the repeat chest HRCT scans six weeks, six months and 12 months after prednisone administration.

A 49‐year‐old man presented to our outpatient clinic on 11 June 2018, complaining of nonproductive cough and exertional dyspnea for less than six months. He had no fever, chest pain or hemoptysis. He was diagnosed with nongerminal center B cell diffuse large B‐cell lymphoma (non‐GCB‐DLBCL, stage III EB) after iliac mass biopsy with colonoscopy, with the involvement of cervical, periaortic arterial, mesenteric and inguinal lymph nodes. After four cycles of chemotherapy with a dose‐adjusted (DA) multiagent chemotherapy regimen, DA‐EPOCH‐R (etoposide, prednisone, vincristine, cyclophosphamide, doxorubicin, rituximab [375 mg/m^2^ per cycle]), the tumor was observed to have almost disappeared on the repeat PET‐CT scan, and his lungs were clear (Fig 1a, 7 February 2018). He tolerated the adverse effects of chemotherapy. To complete his treatment, a fifth cycle of DA‐EPOCH‐R was arranged on 9 February 2018, and he subsequently developed a severe dry cough and shortness of breath on 24 February 2018. A repeat chest X‐ray scan showed diffuse GGOs in the bilateral lungs. The arterial blood gas analysis showed pH 7.43, pCO_2_ 35.3 mmHg, and pO_2_ 42.1 mmHg on room air. Although there was no positive result after testing induced sputum, various empirical antibiotics, including imipenem/cilastatin, moxifloxacin, and sulfamethoxazole‐trimethoprim, were used without effect. His symptoms were ameliorated temporarily after two weeks of methylprednisolone administration (from 5 April to 20 April 2018) during empirical treatment for *Pneumocystis jirovecii* pneumonia (PCP) (160 mg/day × 3 days, 120 mg/day × 3 days, 40 mg b.i.d. × 5 days, 40 mg q.d. × 5 days). His exertional dyspnea had progressed slowly starting one month after discontinuation of the corticosteroid, and he was referred to our clinic. He smoked half a pack daily for approximately 30 years, but he had quit smoking two years ago. There was no palpable superficial lymphadenopathy, no clubbing of the fingers. Scattered velcro‐like crackles could be heard in both basal lungs.

The complete blood count and biochemical panel were almost normal. The erythrocyte sedimentation rate (ESR), high‐sensitivity C‐reactive protein (hsCRP) level, 1,3‐β‐D‐glucan test and galactomannan test were all normal. The 18 item antinuclear antibody (ANA) panel and the test for the antineutrophil cytoplasmic antibody (ANCA) were negative. The DNA detection of cytomegalovirus (CMV) in the peripheral blood was also negative. The pulmonary function test showed restrictive ventilation dysfunction and diffusion impairment: the ratio of the forced expiratory volume in one  second (FEV1) to the forced vital capacity (FVC) was 84.2%, the FVC was 2.99 L (67.3% of the predicted value), and the diffusion capacity of carbon monoxide (DLCO) was 5.99  mmol/minute/kPa (59.1% of the predicted value). A repeat high‐resolution CT (HRCT) scan of the chest showed diffuse bilateral GGOs without obvious lymphadenopathy (Fig 1b, June 2018).

**Figure 2:**
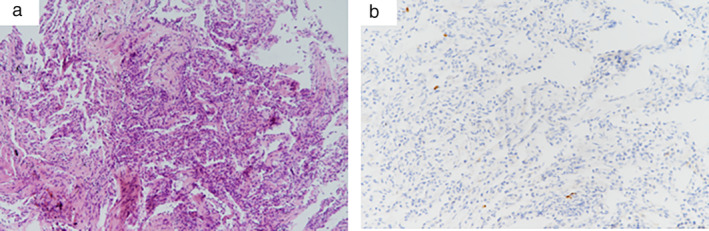
The pathological manifestations of the transbronchial lung biopsy. (**a**) Hematoxylin and eosin staining (magnification, ×100) showed thickened alveolar walls, with fibrous tissue hyperplasia and scant lymphocyte infiltration; and (**b**) immunohistochemical staining showed scattered anti‐CD20‐positive lymphocytes in the lung.

The results of the bronchoscopy were almost normal. The bronchoalveolar lavage fluid (BALF) cultures for bacteria, fungi, mycobacteria and nocardia were all negative. The test for *P. jirovecii* DNA was also negative. The BALF total cell count was 38.2/μL, and the BALF differential cell counts were as follows: 91% alveolar macrophages, 6.5% lymphocytes, 0.5% eosinophils, and 2% neutrophils. T cell subtype analysis of the BALF showed 93.9%, 36.9% and 55.3% for CD3^+^, CD4^+^ and CD8^+^ lymphocytes, respectively. The ratio of CD4^+^ to CD8^+^ lymphocytes was 0.7%. Hematoxylin and eosin staining of the transbronchial lung biopsy (TBLB, magnification, ×100) specimen showed thickened alveolar walls, with fibrous tissue hyperplasia and scant lymphocyte infiltration (Fig 2a). There were scattered anti‐CD20‐positive lymphocytes found on immunohistochemical staining analysis (Fig 2b). The pathological manifestation coincided with the characters of nonspecific interstitial pneumonia (NSIP) pattern.

He was diagnosed with rituximab‐induced interstitial lung disease (RTX‐ILD) after the clinical‐radiological‐pathological specialists' multidisciplinary discussion. Prednisone (0.8 mg/kg/day) was prescribed, his symptoms disappeared, and his pulse oxygen saturation improved gradually over the course of three weeks. The prednisone was tapered gradually (decreased by 2.5 mg per week and then maintained at 10 mg/day). The pulmonary GGOs gradually disappeared on the repeat chest HRCT scans (Fig 1c,d,e, six weeks, six months and 12 months after prednisone administration), and his PFT also improved, with FVC: 3.19 L (72.5% of the predicted value) and DLCO: 6.87 mmol/minute/kPa (68.2% of the predicted value).

## Discussion

The combination of rituximab (RTX) and chemotherapy improved both response rates and survival outcomes compared with chemotherapy alone for DLBCL patients without causing significant additional treatment‐associated adverse events (AEs).^1^ Keefer *et al*.^2^ retrospectively analyzed the respiratory complications of RTX‐containing chemotherapy for patients with non‐Hodgkin's lymphoma (NHL): 24% of cases developed respiratory complications, and various pulmonary infections (75%) were the most common event. However, pulmonary lymphoma infiltration and other kinds of noninfectious pulmonary complications might also be life‐threatening, eg, RTX‐ILD^3^, ^4^ and chemotherapy‐associated cardiotoxicity leading to pulmonary edema.

Although three different clinical phenotypes of RTX‐ILD have been reported, including the early onset hyperacute phenotype, delayed onset acute phenotype and late onset chronic phenotype, the delayed onset acute form is the most common.^3^ This type of RTX‐ILD usually occurs two weeks after the fourth cycle of RTX therapy for lymphoma. Middle‐aged male patients more commonly develop this complication, and the combination of RTX with chemotherapy is more likely to lead to the development of RTX‐ILD than is RTX monotherapy.^4^, ^5^ Acute/subacute hypoxemic dyspnea and dry cough are the main clinical manifestations, which are usually accompanied by fever in the early stage. Most patients recover with the early administration of corticosteroids.^3^, ^4^, ^5^ However, approximately 15% of patients with RTX‐ILD die from it.^4^ The onset of RTX‐ILD in our patient occurred two weeks after the fifth infusion of RTX when he complained of progressive exertional dyspnea and nonproductive cough, which are the typical manifestations of RTX‐ILD. These symptoms improved temporarily with the administration of corticosteroids and gradually worsened after they were discontinued.

The pathogenesis of RTX‐ILD is not as yet well elucidated. Several speculations have been raised as follows:^4^ (i) Prolonged B cell depletion by RTX could lead to the increased activation of cytotoxic T lymphocytes and subsequent lung damage; (ii) release of abundant inflammatory cytokines and cytotoxic substances to the pulmonary circulation during the RTX‐containing chemotherapy. However, RTX has been tried successfully for kinds of connective tissue disease (CTD), especially for severe or refractory CTD associated ILDs and non‐idiopathic pulmonary fibrosis ILD recently.^6^, ^7^ RTX‐ILD has been reported to be more common in B cell lymphoma than in CTD.^4^ However, the mechanism is not well understood. Well designed large‐scale clinical trials and studies are expected.

Chest HRCT scan is the fundamental and most important examination for all types of ILDs. Multiple focal alveolar densities in combination with diffuse GGOs are the most commonly reported chest CT manifestations of the delayed‐onset acute form of RTX‐ILD.^3^ However, only diffuse bilateral GGOs were observed in our patient at our hospital, which meant that opportunistic pulmonary infections, eg, PCP, CMV, and fungal infections had to be ruled out. PCP has been reported as a result of combination therapy with RTX and chemotherapy, and prophylaxis for PCP is recommended.^8^, ^9^ In our patient, the pathogen examinations of the BALF and/or blood were negative for all suspected pathogens, and the empirical antibiotics were ineffective. RTX‐ILD was strongly considered as a diagnosis in our clinic.

Lung biopsies cannot be performed routinely for all suspected cases of RTX‐ILD. For those who undergo lung biopsy, organizing pneumonia is the most commonly reported pulmonary pathological pattern, whether alone or in combination with nonspecific interstitial pneumonia (NSIP) or usual interstitial pneumonia (UIP).^3^, ^10^ This patient's TBLB features indicated the NSIP pattern,^11^ and this is probably the first report of a case of an isolated NSIP pattern of RTX‐ILD. There was no significant indication for pulmonary infectious diseases. CD20 is a surface antigen that is exclusively expressed by B‐lymphocytes, including malignant and normal B cells. CD20‐positive malignant B cells will be abundantly infiltrated in the lung for pulmonary B cell NHL.^1^ In our patient, pulmonary lymphoma infiltration could be excluded based on the immunohistochemical staining of scattered anti‐CD20‐positive lymphocytes in the alveolar septum.

In conclusion, RTX‐ILD might be considered in the differential diagnosis of patients treated with RTX, especially if a patient is nearing the time of administration of a fourth cycle of RTX. However, the pathogenesis of RTX‐ILD is not fully understood. A definitive diagnosis of RTX‐ILD should be made based on the combination of clinical, chest HRCT and pathological examinations. The early administration of corticosteroids might be effective after a diagnosis of RTX‐ILD is made.

## Disclosure

No authors report any conflict of interest.
